# Do you share your personally useless information if others may benefit from it?

**DOI:** 10.1371/journal.pone.0276062

**Published:** 2022-10-17

**Authors:** Aryan Yazdanpanah, Abdol-Hossein Vahabie, Majid Nili Ahmadabadi

**Affiliations:** 1 Cognitive Systems Laboratory, Control and Intelligent Processing Center of Excellence (CIPCE), School of Electrical and Computer Engineering, College of Engineering, University of Tehran, Tehran, Iran; 2 Department of Psychology, Faculty of Psychology and Education, University of Tehran, Tehran, Iran; 3 School of Cognitive Sciences, Institute for Research in Fundamental Sciences, Tehran, Iran; St John’s University, UNITED STATES

## Abstract

Information is personally useless if its beholder cannot individually benefit from it further unless she shares it with those who can exploit that information to increase their mutual outcome. We study sharing such information anonymously in a non-strategic and non-competitive setting, where selfish and cooperative motives align. Although sharing information was cost-free and resulted in expected mutual payoff, almost all subjects showed some levels of hesitancy toward sharing information, and it was more severe in the introverts. According to our mechanistic model, this irrationality could arise because of the excessive subjective value of personally useless information and low other-regarding motives, that necessitated over-attainable personal benefit to drive sharing. Interestingly, other-regarding element correlated with the subjects’ belief about how others are cooperative in general. In addition, sensitivity to the value of information correlated with their extraversion level. The results open a new window towards understanding inefficient motives that deprive people of collective benefit.

## Introduction

Information sharing is ubiquitous and with the rise of social networks and email lists, it is becoming less costly (500 milion active users and 15 bilion messages delivered daily in Telegram, 2 bilion Instagram users, 6000 tweets every second, and 500 milion tweets per day) [1–4]. For example, people can retweet a post, forward an email, share an Instagram story, a WhatsApp, or a Telegram message with others just by tapping on their phones. There are many situations in which the information itself is not beneficial for the information beholder (personally useless information), but it can be useful for others. For example, imagine you see a Ph.D. position, a sale announcement, or a job offer on Twitter that can be helpful for your friends or nonfriends but not for you. This personally useless information can be used by the circle of our friends and makes a better public image of us, or it can be exploited by many anonymous people who probably will not remember our names some months after using our shared information (e.g., seeing our tweet as a suggested post and using our information). What do you usually do in these sorts of situations? What are the psychological constructs of sharing information with others who will not impact our social identity and relational dynamics?

Our information-sharing strategy is complex and multifactorial. For example, Wittenbaum et al. [5] showed that information sharing is a motivated process in a way that group members decide what information to be shared and how to mention it to particular members of the group. People also might inform others in order to present themselves, i.e., as a form of discussing their own traits, preferences, and emotions while it activates regions associated with reward in the brain [6, 7]. Another possibility is that people share information to display their prosociality or trustworthiness [8–10]. In addition, individuals can share information to improve others’ well-being [11] or simply because they enjoy others’ good fortune [12, 13]. Scholz et al. [14] proposed a parsimonious model of sharing behavior that is called value-based virality. In their suggested model, self-related motives and social values play a key role in forming the value of sharing information; we share information to show a positive image of ourselves and expect a social reward by sharing the information that we share. Tamir et al. [15] claimed that information sharing is intrinsic and is associated with reward circuits in the brain, even in costly situations and where people are anonymous and altruistic motives disappear. In their study, shared information did not have any benefit for the receivers. Therefore, taking the mentioned factors into account, one may speculate that people should exhibit a higher tendency for sharing when receivers can potentially gain monetary benefit from that information, even if the sender has no benefit in that. Furthermore, we study sharing information anonymously because with the rise of social media and digital platforms, there are lots of situations in which people can share information anonymously (e.g., disseminating information in mailing lists or social media that our non-friends can also benefit from it and does not affect our reputation). Also, studying sharing information anonymously, helps us to investigate the role of pure altruism in absence of public-image gain.

Obviously, sharing information is counter-optimal in many cases. For example, when the environment is strategic and competitive, sharing information worsens the payoff [16, 17]. The cost of communication also negatively affects information-sharing behavior. Duffy et al. [18] showed that the communication cost reduces the “size of the language”, meaning that the subjects tend to transmit fewer elements in the messages. Competition and limited resources seem to influence the information sharing intentions as well negatively.

In the present study, we investigated personally useless information sharing behavior when the information giver and receiver could gain monetary benefit from it. In order to dissociate the confounding effect of the receiver type, we made the information givers and receivers anonymous to each other. However, the information receiver was present in the room to make the information givers feel that they are playing with a real person. Furthermore, we studied subjects’ sharing behavior in a controlled condition that the task was non-strategic and non-competitive, resources were unbounded, and information sharing was non-costly to remove the proposed barriers for sharing. Furthermore, sharing information increased expected payoff of receivers, and subjects can gain benefit from that payoff. By means of behavioral experiments, computational modeling, and Big Five personality traits we investigated four possible mechanisms by which people might share their personally useless information while it could be financially beneficial for others as well as themselves:

1. One can hypothesize that people do not share their personally useless information since they think it is not fair to provide an opportunity for someone else while they have not had the same opportunity [19].
2. People might take fairness of outcomes considerations into account when they share information [20, 21]. In this view, subjects’ hesitation to share information is not because they think the opportunities are not fair. Instead, the main barrier for sharing is because they think the outcomes are not fair.
3. The third explanation relates self-regarding and other-regarding motives to sharing the information that may result in money [22]. In this view, individuals’ hesitation in sharing information does not occur because they think the opportunities or outcomes are not fair, rather, it happens since they do not want to help others or even themselves because they have low other-regarding or self-regarding motives, respectively. Note that while in many physical situations in the real world, people have to choose between themselves or others (selfishness and prosocial behavior result in the opposite directions), the subjects’ selfish and other-regarding motives are not contradictory in general, especially in the digital world. For example, one could have a high self-regarding motive while at the same time caring a lot for others as well (high other-regarding motive). We designed our experiment in a way to be capable of dissociating the roles of self-regarding and other-regarding motives.
4. Another explanation for sharing personally useless information might be related to the level of cooperation or subjects’ beliefs about others’ cooperation [23, 24].

Furthermore, we would like to investigate whether sharing personally useless information is related to personality traits or not. Particularly, we considered extraversion as a trait that might facilitate information-sharing behavior since extroverts are more willing to communicate with others [25].

## Methods

**Fig 1:**
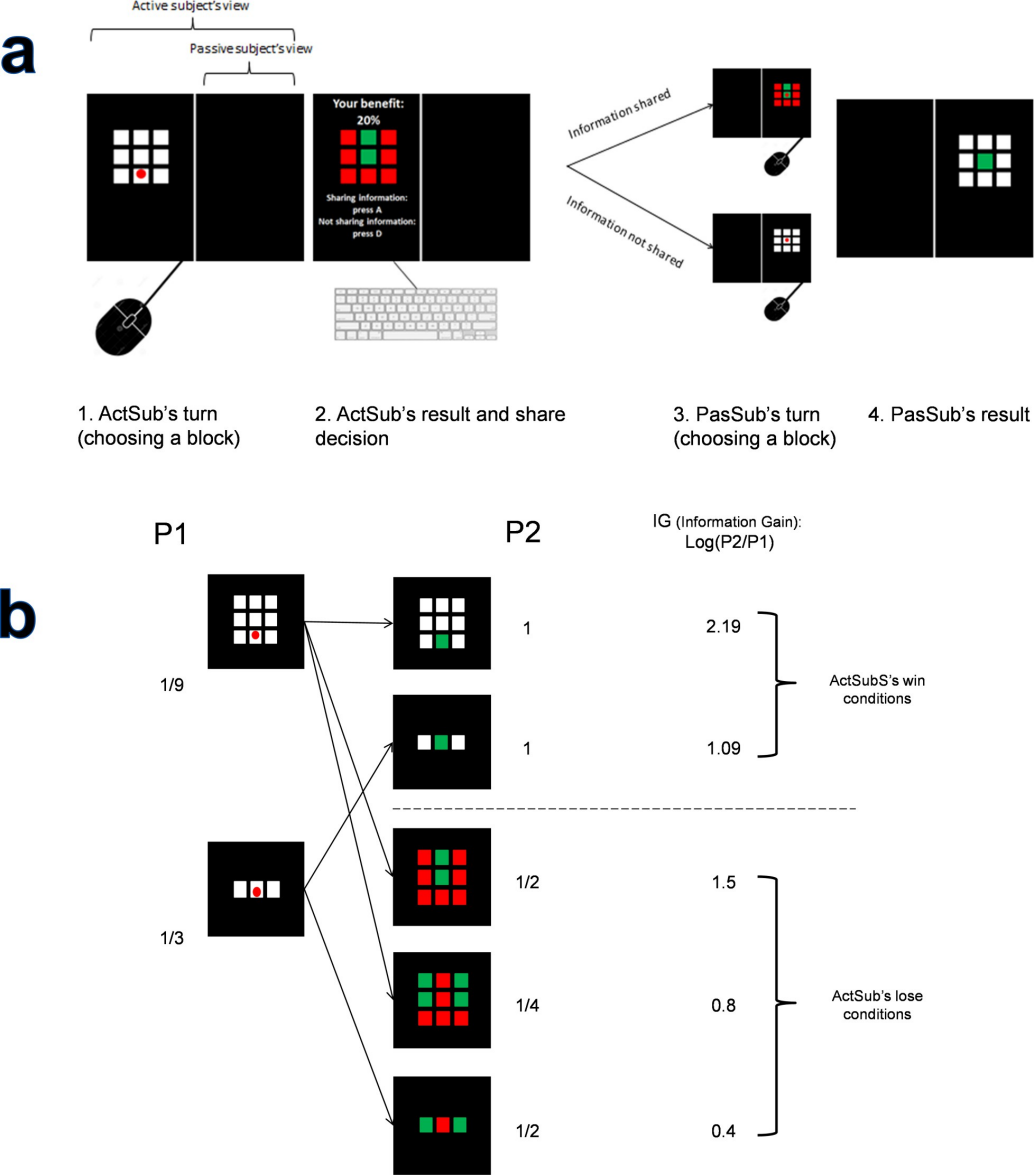
a) The trend of the game for one trial is shown. ActSub can see the whole screen, but PasSub can only see her own side. We changed the side of the PasSub for every new subject. 1. ActSub has to choose a white square with a mouse. 2. ActSub result is shown to her, and some information is delivered to her as a result of her choice. At this time, she is asked if she wants to share her information. She will take this decision by pressing one of the two keyboard buttons (D or A). These buttons changed for shared and not-shared decisions for every two subjects so that the PasSub side and the buttons had all four possible combinations. The percentage of benefit is also shown in this state. We wrote all of the texts in the Persian language. In this example, the percentage of benefit is 20% (درصد سهم: ۲۰٪). The squares were smaller in the original experiment, and we magnified them here for better visualization. 3. PasSub has to choose a square with a mouse. If ActSub decides not to share her information, PasSub will play the same game that ActSub did and has to choose a square from one of the white squares. Otherwise, If ActSub shares her information, PasSub will play the game with the new information that ActSub has acquired and has to choose a square from one of the green squares. 4. PasSub result is shown. If the PasSub chooses the right square, PasSub will succeed and earns 100 scores. If the percentage of benefit is more than zero, ActSub will take that amount from 100 scores. In this example, ActSub will take 20 scores, and PasSub will take 80 scores out of 100. Every 100 scores are 10000 Rials of Iran’s currency. Otherwise, PasSub will fail, and none of them will earn anything from PS’s choice. b) The probability of a win for ActSub is always 1/9 or 1/3 in each trial. If ActSub does not share her information, the probability of a win for PasSub will also be 1/9 or 1/3, respectively. If ActSub shares her information, the probability of a win for PasSub will be one of the five conditions depicted in the figure.

To answer these questions, we designed a two-player game (see Methods) in which only one of the players could always share her information (Active Subject or ActSub). The other player was passive in all of the trials and could not share information at all. All 70 subjects played the role of ActSub, and the research assistants in the lab played the role of passive subjects (PasSub). PasSub used the information that was provided by the ActSub in each trial. ActSub and PasSub could not see each other and could not talk to each other before, during, or after the task, but they were in the same room but could hear each other’s playing (clicking on the mouse or pressing a key on the keyboard). We made a cover story to make the subjects believe that they have been randomly assigned to their roles. ActSubs were told that both the information giver (ActSub) and information receiver (PasSub) would be paid at the end of the experiment based on the score of 25 randomly chosen trials for each of them. However, PasSubs were actually the lab members and were not identifiable by the ActSubs due to having a divider between them. PasSubs were actually compensated financially in another way regardless of their score. Each trial began with ActSub’s turn, and some white squares (9 or 3) were shown on the screen that only one of them had a reward (Fig 1A). The trials were independent of each other, and the place of the reward changed in each trial randomly. After choosing a square, ActSub would either win or lose. In “win” conditions, ActSub acquired the exact location of the reward and could report this information to her confederate (Passive subject or PasSub). However, in the “lose” conditions, ActSub was given some information about the possible places of the reward that could share with PasSub. Therefore, in the “lose” conditions, she could increase the PasSub’s reward probability by sharing information, but she could not make it 100%; see Fig 1A and 1B. If ActSub decided to share her information, ActSub would either take a benefit (20% or 50%) from PasSub’s current trial reward (if the PasSub won, both would take a benefit. Otherwise, none of them would benefit from sharing information) or would take nothing from sharing information (0% benefit). ActSub or PasSub could not determine the benefit percentage, and the amount of benefit was determined by the experimenter in each trial and was randomly chosen from one of the three benefit percentages (0%, 20%, or 50%).

### Participants

Seventy-one students from the University of Tehran (34 females & 37 males, aged between 18–32 years old with a mean of 22.5) participated in the experiment. After the task, we asked participants some questions to ensure that they have correctly understood our procedure. One male subject was excluded after this part since he had not understood the task. The subjects were paid 150K Rials for participation (almost $4 at the time of the experiment was taken), and an additional payment ranged between 150K-300K Rials. The additional payment was their score in 20 randomly chosen trials. Informed consent was obtained from all subjects. The study was approved by the ethics committee of the University of Tehran, and the experiments were carried out in accordance with relevant guidelines and regulations.

### Task

The experiment consisted of 250 trials. Subjects were interviewed after the task to talk about their strategies and to make sure that they had understood the task correctly. Prior to the experiment, subjects were provided a written instructions about the task. After reading the instruction, subjects played a training phase consisting of 5 trials and asked questions about the task after this phase. In the game, using a mouse, ActSub had to choose one of the 3 or 9 white squares presented on the screen. Only one of the squares was associated with reward (Fig 1A).

Subjects were aware that the place of the reward changes in each trial randomly. ActSub could observe the PasSub’s screen, but PasSub could not see ActSub’s screen, and ActSub was aware of that (Fig 1A). Contrary to the dictator game in which people are given an amount of money, people obtained information by themselves in our task. If the ActSub selected the rewarding square, she would win, and the color of the chosen square would change to green. Otherwise, the ActSub would fail, and as a result, the selected square, as well as some other non-rewarding squares, would become red, and the remaining squares would turn green. The green squares meant that the reward was behind only one of them.

At this time, the ActSub was asked to push Key A if she decided to share the acquired information with the PasSub (called shared-condition), in return for a percentage of benefit (0%, 20%, or 50%, which was shown on the screen) of PasSub’s possible gain, and to push Key D otherwise (called non-shared-condition). The keys were counterbalanced between subjects. We call this number ‘ActSub’s benefit percentage,’ and it means that the ActSub gains benefit from her confederate’s score in that specific trial if the ActSub gives the information to the PasSub and PasSub succeeds as a result of that. In the shared condition, PasSub had a higher probability of a win compared to ActSub, and in the non-shared situation, the PasSub would play the original game.

Depending on how many white squares would turn to green, we had three different conditions for nine squares trials and two different conditions in three squares trials. Fig 1B illustrates these different conditions. The probability of a win for ActSub (*P*_1_) was always 1/9 or 1/3 in each trial, and PasSub had the same win probability in non-share conditions. In the shared condition, PasSub’s win probability (*P*_2_) would be 1, 1/2, or 1/4, depending on how many squares had turned to green. We define information gain (IG) as log(P_2_/P_1_) for shared-condition; see Fig 1B. We had three different ActSub’s benefit percentages (0%, 20%, and 50%) and five different information gains, summing up to 15 different decision conditions for the ActSub.

## Results

A naive assumption is that the subjects in our task always share their personally useless information since sharing information imposes no cost and may result in income for them in 66% of trials. Sharing information in all trials reduces the decision process load as well. The question is what percentage of the subjects follow the naive assumption and what is the information-sharing strategy for those who do not follow it.

### Role of win and lose

For a pure reward maximizer, the sharing strategy should be invariant with respect to the history of the subject’s gain in the current trial. To test this, we calculated the average frequency of sharing in the win and lose conditions. The subjects were significantly less generous in sharing when they gained self-exploited full information; average sharing was 37% (-/+0.038) against 55% (-/+0.033) in uncertain information cases (two-sided paired t-test, t-stat = -6.92, P-value <1e-8, significance level = 0.05, between subjects analysis).

### The sensitivity of ActSub to her benefit percentage

If subjects want to minimize their decision load, they must be extremist; they either share or do not share in all trials. Analysis of sharing frequency of all subjects revealed that their average sharing is 26% (-/+0.037) when their benefit is zero. Therefore, decision load minimization is not the case, at least for all subjects. The ratio of information sharing frequencies in 50% and 0% benefit cases is in favor of labeling the subjects as partially gain seekers; their average sharing is 74% (-/+ 0.033) in 50% benefit cases while it is 26% in 0% benefit condition (two-sided paired t-test, t-stat = 11.88, df = 69, P-value <1e-17, significance level ~ 0.01 with Bonferroni correction, between subjects analysis). Interestingly, in the 50% benefit case, when the subjects have a fully fair percentage of their partner’s reward, they do not always share their personally useless information. In 20% benefit case, the subjects share information in 54% (-/+0.043) of trials, which is significantly lower than the same statistic in 50% benefit condition (two-sided paired t-test, t-stat = -6.05, df = 69, P-value <1e-7, significance level ~ 0.01 with Bonferroni correction, between subjects analysis). This observation is against assuming the subjects as pure gain seekers. The subjects shared information further less in 0% benefit condition in comparison with 20% condition, (two-sided paired t-test, t-stat = -8.66, df = 69 P-value <1e-11, significance level ~ 0.01 with Bonferroni correction, between subjects analysis). Contrary to the naive assumption, the results indicate that the subjects’ utility function for information sharing is not a pure income maximization while information sharing does not have a cost. The task is neither cooperative nor competitive and sharing results in probable income in 2/3 of the cases.

The question which arises here is why subjects do not share their personally useless information and what drives this dissociation in their behavior between different benefit percentages?

### Level of cooperation and tendency to share information

One possible reason that one might share her information in our setting is the level of cooperation, i.e., the more subjects cooperate in a public good, the more they are willing to share their personally useless information. In order to investigate this possibility, we took a public goods game (PGG) study from 30 subjects (from various ranges of sharing personally useless information) who participated in our first task. The hypothesis that sharing personally useless information is related to the level of subjects’ cooperation was ruled out, Fig 3A, (Pearson correlation, R = 0.12, p-value = 0.50). Furthermore, we analyzed the relation between sharing information in each benefit percentage (and also the difference between sharing in benefit percentages), with the level of subjects’ cooperation in PGG. Again, we did not observe any significant correlation between the frequency of sharing in any of the benefit percentages and the level of cooperation in PGG.

Another explanation for not sharing personally useless information is the subjects’ beliefs about the level of others’ cooperation. To test this, we recorded the subjects’ beliefs about the levels of three other participants’ cooperation in the PGG and used its mean, which we call it “average belief about others’ cooperation.” The results indicate that there is no significant correlation between the “average belief about others’ cooperation” and the tendency to share information in our first task, Fig 3B, (Pearson correlation, R = 0.17, p-value = 0.36).

However, by analyzing the relation between the “average belief about others’ cooperation” and the tendency to share information in each benefit percentage, we saw that there is a significant correlation between the tendency to share information in 0% benefit and the “average belief about others’ cooperation,” Fig 3C, (Pearson correlation, R = 0.39, p-value = 0.03). It means that there is a relation between the subjects’ belief about how much others are cooperative toward them and giving free information to a confederate in the information-sharing task.

In addition, there is a negative correlation between their “average belief about others’ cooperation” and the difference between sharing information in 50% and 0% benefit (Pearson correlation, R = -0.49, p-value = 0.005). It means that the more subjects want to take benefit from their confederate, and the less they want to give free information to them, the less they think others cooperate in PGG.

### Fairness of opportunity or information gain (IG) and the tendency to share information

The information gain (IG) varies from 0.4 to 2.19 in our task (see Fig 1) and can also be seen as the fairness of opportunity since there is a difference between the probability of winning for ActSub and the probability of winning for PasSub if the Actsub decides to share. Therefore, if a subject is sensitive to the opportunity given to him relative to the opportunity provided for another person, the subject has to share less if the IG gets larger. We calculated the average frequency of sharing (we call it the tendency of sharing information) in each information gain condition. In lose conditions (IGs: 0.4, 0.8 and 1.5), there is not a significant difference between average frequency of sharing in different IG conditions (one way ANOVA, F-stat = 0.14, df = 2, p-value = 0.86, between subjects analysis). Besides, the task complexity for ActSubs, which is 3 or 9 square cases, does not affect the subjects’ sharing behavior when they succeed; i.e., the sharing frequency is 37% in both win conditions (two-sided paired t-test, t-stat = -0.40, P value = 0.68, significance level = 0.05, between subjects analysis).

### Personality traits and the tendency to share information

Another possible explanation for sharing information might be related to the subjects’ personality traits. Our analysis showed a positive correlation between sharing information and extraversion, i.e., the more extraverts the subject is, the more she is inclined toward sharing personally useless information (Pearson correlation, R = 0.29, p-value = 0.027). However, there was not any significant correlation between the tendency to share information and any other Big Five factors.

### GLM model on sharing information

In order to analyze the results further, we fitted a GLM model to our task structure. We analyzed subjects’ sensitivities to different conditions in our task (for further reading, see S1 File, GLM model). Three key factors could potentially influence subjects’ behavior in our task: (i) benefit percentage, (ii) The Information Gain (IG=log(P(PasSubwins|infoshared)/P(PasSubwins|infonotshared))) which is the increase in the probability of PasSub’s winning in the condition that ActSub decides to share relative to the condition that ActSub decides not to share, (iii) “win” condition. Table 1 shows the subjects’ mean and standard deviation of the fitted parameters. Also, it represents what percentage of subjects have negative/positive sensitivity (beta coefficients) for each variable in our GLM model (see S1 File, GLM model, for the detail of the parameters and standardized coefficients regarding the calculating significant parameters).

**Table 1:** Parameters of the GLM model for each subject and percentage of positive, negative, or nonsignificant beta coefficients among subjects.

Parameter	mean	95% CI	Positive beta coefficient	Not significant beta coefficient	Negative beta coefficient
*β*_0_ (intercept)	-5.49	[-12.56, 1.56]	~14%	~36%	~50%
*β*_1_ (benefit percentage)	0.30	[0.12, 0.48]	~75%	~25%	0%
*β*_2_ (information gain)	-0.23	[-0.44, -0.02]	~7%	~76%	~17%
*β*_3_ (win)	-6.12	[-12.00, -0.25]	~2%	~58%	~40%

**Fig 2:**
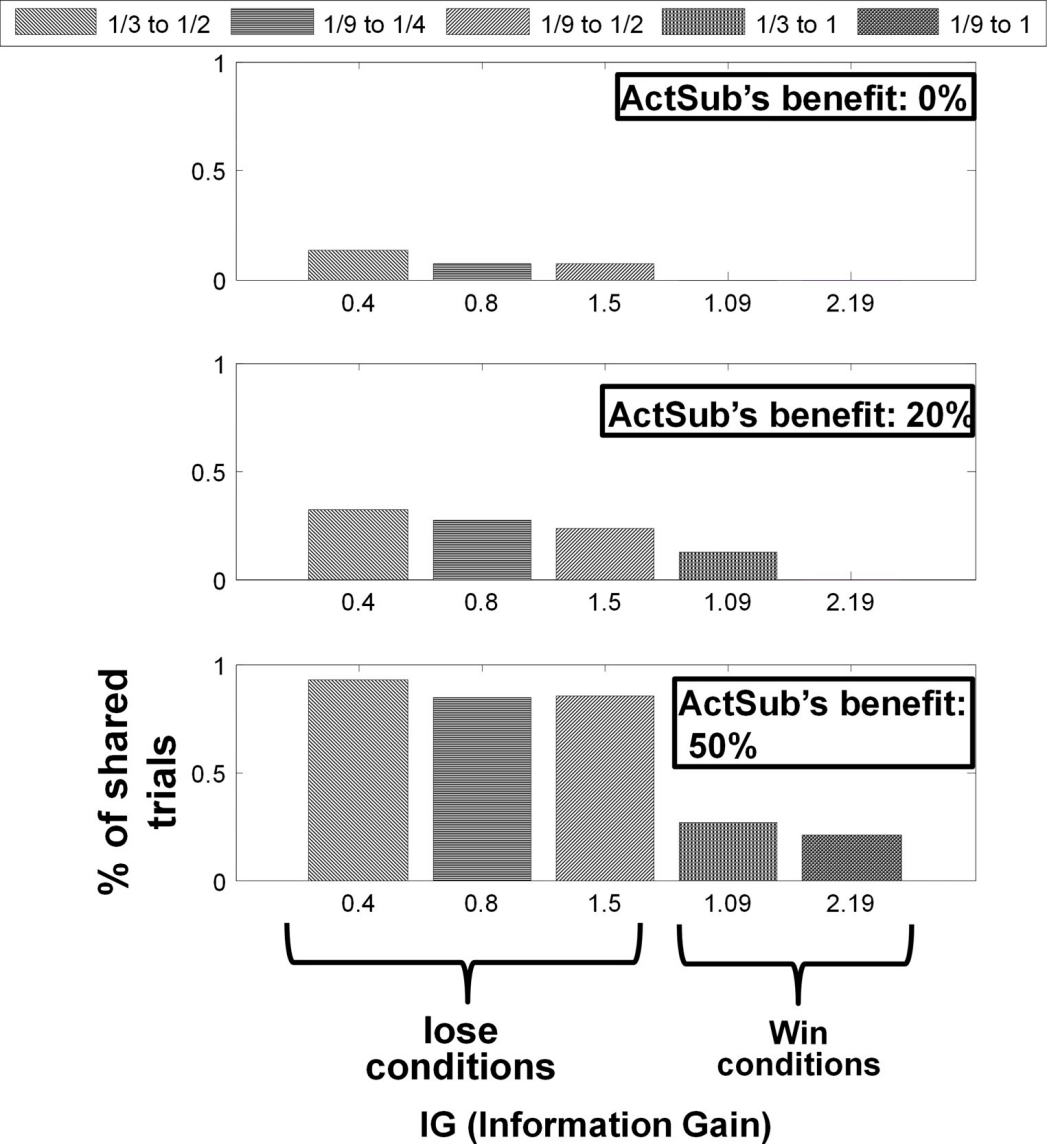
The average frequency of sharing responses is shown with details in the pool of data. We had three different benefit percentages for ActSub (0%, 20%, and 50%), and for each benefit percentage, we had five different IGs. Two of these IGs are win conditions, and 3 of them are the conditions in which the ActSub has failed. The IGs are sorted for the win and lose conditions separately. Standard errors of sharing frequencies among the subjects are depicted in each of the 15 conditions.

**Fig 3:**
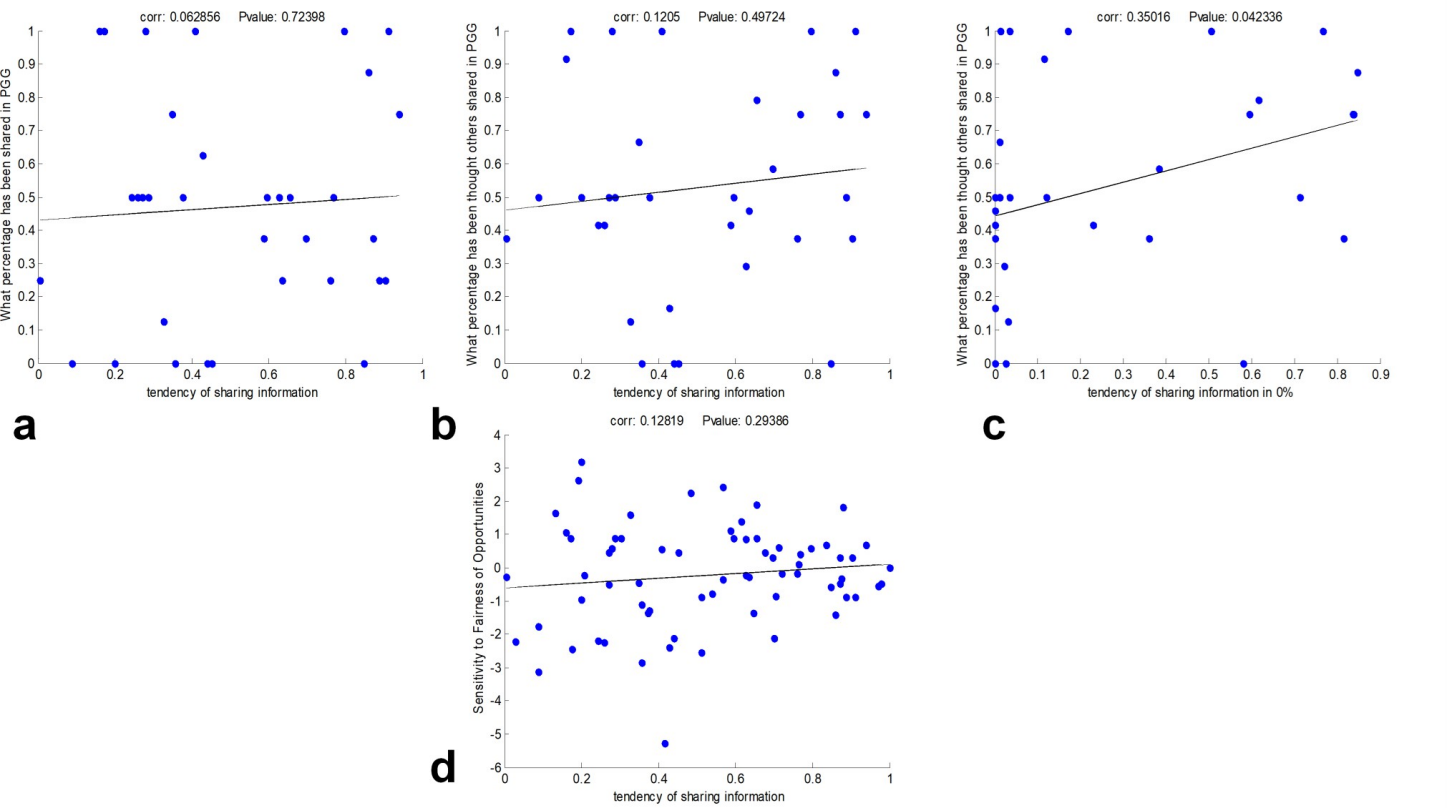
Information sharing and behavioral factors a) tendency of sharing information and subjects cooperation in Public Goods Game (PGG) b) tendency of sharing information and subjects’ beliefs about others cooperation in PGG c) tendency of sharing information in zero benefit and subjects’ belief about others cooperation in PGG d) tendency of sharing information and sensitivity to the fairness of opportunities.

As in Fig 2 (which is the mean frequency of shared trials in each condition of the task) and as the GLM model describes (Parameter *β*_1_), a considerable number of subjects have a negative tendency toward sharing personally useless information, and some subjects do not follow the naïve assumption discussed earlier. Furthermore, we found no correlation between the sensitivity to IG or fairness of opportunities (GLM model parameter *β*_2_) and the tendency to share information, Fig 3D.

In another series of retrospective analyses, we investigated the role of the previous trial’s envy, guilt, and regret on subjects’ decisions. We defined guilt as an emotion that arises after ActSub decides not to share and the PasSub does not win in the same trial (i.e., the ActSub might think that if s/he had shared the information, the PasSub would not have lost and therefore feels guilty for not sharing). Also, ActSub might feel regretful if s/he decides not to share and the PasSub wins. Or might feel envious if the PasSub’s payoff has been more than ActSub’s payoff in the previous trial (look at the S1 File for more details). The analyzes showed that these factors were not responsible for the sharing decision (sensitivity to retrospective guilt and the tendency to share information: Pearson correlation, R = 0.19, p-value = 0.10, sensitivity to retrospective regret and their tendency to share information: Pearson correlation, R = -0.13, p-value = 0.29, retrospective envy and sharing personally useless information: Pearson correlation, R = -0.08, p-value = 0.50).

### A mechanistic approach to subjects’ information-sharing tendencies

Here, we use two famous social preference models to explore the mechanism by which subjects tend to share their information.

One might assert that fairness of outcomes forms people’s decisions toward sharing personally useless information, i.e., the subjects prefer the 50% benefit percentage. To test this hypothesis, we fitted the Fehr-Schmidt [20] model. In this model, the information giver’s utility function reduces if her confederate’s gains more than her by sharing information (prospective envy), or the subject’s outcome becomes more than her confederate’s outcome by sharing information (kindness, if the subject has negative sensitivity to this condition).

On the other hand, one can simply reject the fairness of the outcome hypothesis and proclaim that subjects’s self and other-regarding behavior [23] can influence sharing personally useless information that can have monetary benefits for them and others. To test these two competing hypotheses, we fitted these two models to our data and compared them to each other (for further reading, see S1 File, computational modeling).

**Fig 4:**
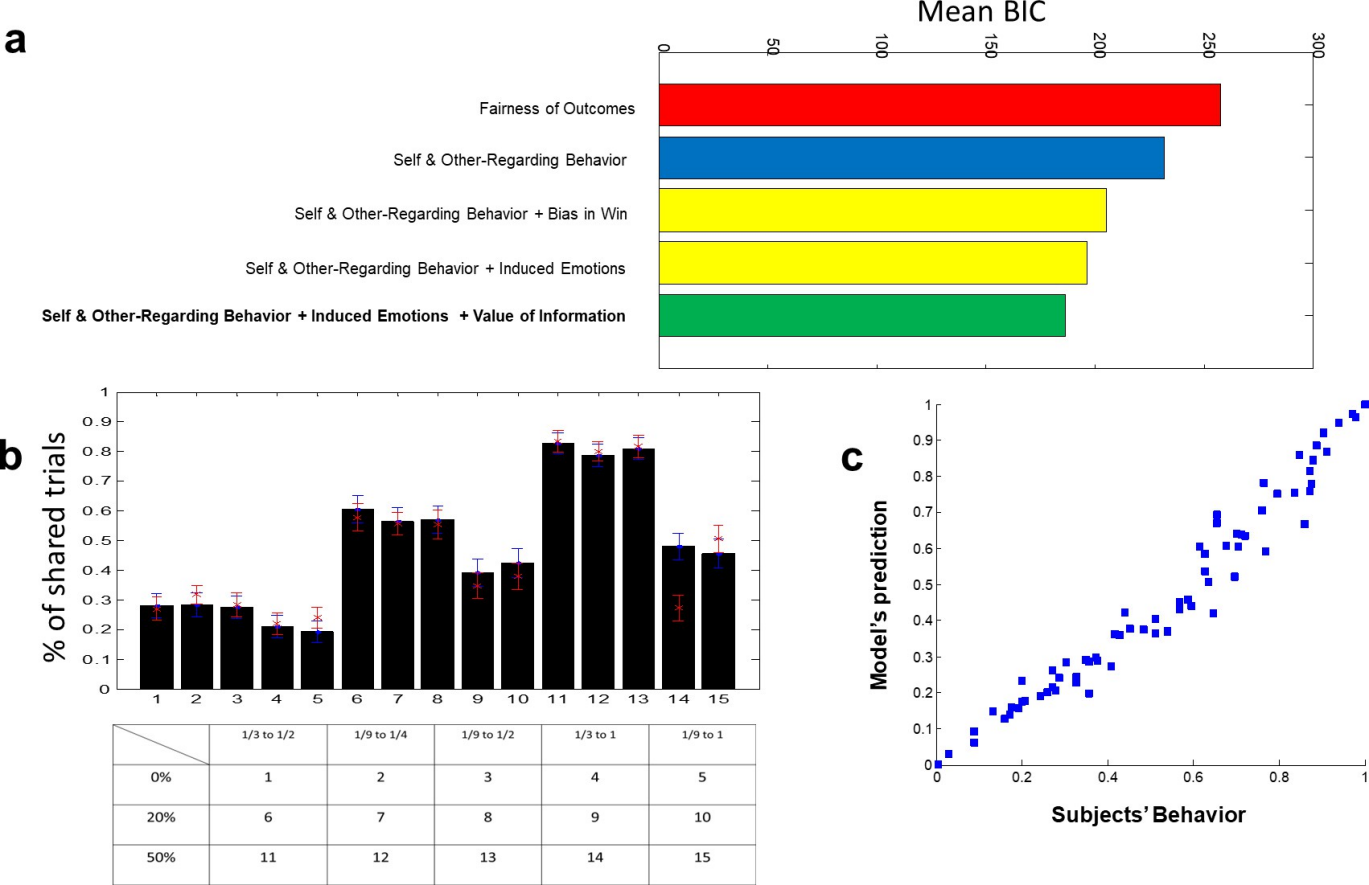
Model comparison and model recovery is depicted a) Mean BIC of models have been compared. The first model from above (the red line) is the Fehr-Schmidt model that is based on the fairness of outcomes. Kindness or envy drive subjects’ decisions in this context. The next model (the blue line) is Hutcherson et al. model that assumes subjects consider their opponent as well as their payoffs to take social decisions. Thus, self and other-regarding behavior drive subjects’ decisions in this context. The remaining three models (yellow ones and the green one) are based on the Hutcherson et al. model. Both of the yellow models take the “win” condition into account. The first yellow model from the above assumes that the “win” condition influences subject decisions separately. However, the second yellow model assumes that the “win” condition changes their social preferences. The last model (the green line) that is the winning model, is similar to the second yellow model, except it assumes that subjects take the value of information into account, i.e., if information has a higher probability of winning compared to other information, it has a higher value. b) Model simulation in the 15 conditions of the task. Red dots and error bars (SEMs) indicate model simulations, whereas black bars with blue error bars indicate subjects’ behavior. c) Predicted behavior of subjects vs. real behavior of subjects.

The mean BIC of these two models is depicted in Fig 4A (red and blue lines). The self and other-regarding model can capture sharing behavior better. However, the self and other-regarding concepts did not have a good prediction solely. Therefore, we used three other variants of the model (yellow lines and green line in Fig 4A) based on the observed behavior of the subjects and the literature of information value. The two yellow models are the self and other-regarding models plus change in the utility function in the “Win” condition that was mentioned earlier. We can add the “Win” conditions in two different ways. First, we can assume that the “Win” condition affects the sharing process independently, and therefore, it can be added as a bias to the model (the upper yellow line in Fig 4A). The second way is to assume that subjects’ self and other-regarding motives change in the “Win” condition. (lower yellow line in Fig 4A). This second proposition had a better model fitting index, so we proceeded with this model. The last parameter that is consistent with our observed data and the literature is what we call “value of information.” As we mentioned earlier, subjects’ sharing tendency positively correlates with their level of extraversion. Furthermore, if we assume that information has intrinsic value in people’s minds [26], we can add the “value of information” parameter to the model. We did this by adding the winning probability if the information giver decides to share the information (*p*_2_ in Fig 1B), that is, the information that the subject can provide for the information receiver. In this context, the more *p*_2_ is, the more becomes the winning probability for the information receiver and thus the information becomes more valuable (we also used other variants of this variable in the model, but this variable had the best prediction and fitting results).

We also used two different models from the literature, Bolton-Ockenfels [27] and Krajbich et al. [28] that did not have good predictions in comparison with the fairness of outcomes and self and other-regarding models. For further reading, see S1 File, computational modeling.

Thus, the proposed model for sharing personally useless information is the combination of self and other-regarding behavior plus changes in these parameters in the “Win” condition as well as the value of information. Fig 4B and 4C show the model recovery part for this proposed model in each of the 15 conditions and in all of the trials, respectively. The proposed model prediction is good in general as well as almost in each of the conditions (except condition 14 because of lack of data).


$$U(share)={(W}_{self}+W_{self\_change}\times Win)\times {Payoff}_{self}(share)+{(W}_{other}+W_{other\_change}\times Win)\times {Payoff}_{other}(share)+W_{Inf}\times P_{2}$$ (1)

$$U(Not\;share)={(W}_{self}+W_{self\_change}\times Win)\times {Payoff}_{self}(Not\;share)+{(W}_{other}+W_{other\_change}\times Win)\times {Payoff}_{other}(Not\;share)$$ (2)

Pr(share)=exp(U(share)/τ)/(exp(U(share)/τ)+exp(U(Notshare)/τ)) (3)

**Fig 5:**
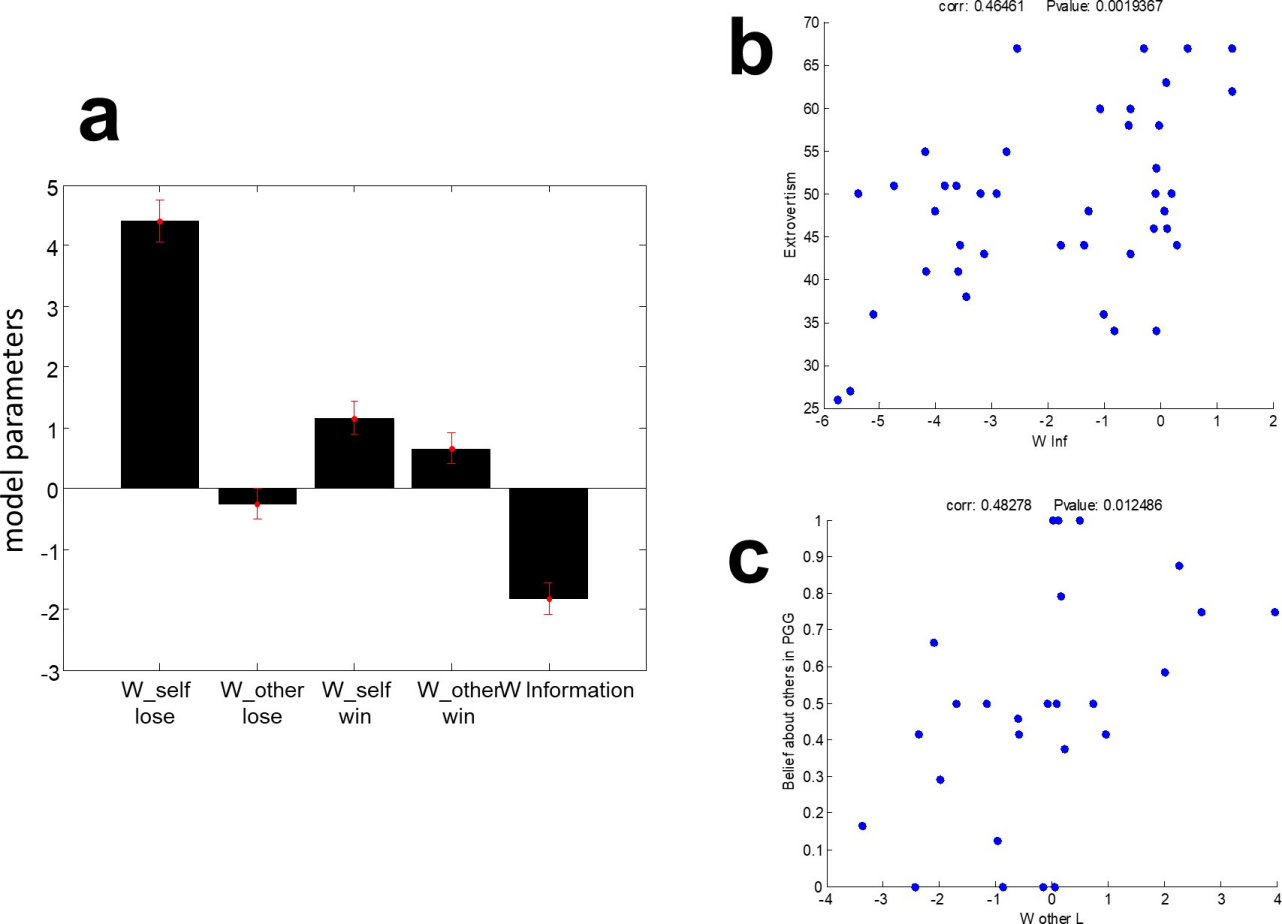
Proposed model parameters’ mean and SEM plus correlation with PGG and Big Five personality traits. a) mean and SEM of model parameters. Note that ***w***_***self*_*win***_ = ***w***_***self_lose***_+***w***_***self_change***_ and ***w***_***other_win***_ = ***w***_***other_lose***_+***w***_***other_change***_. b) there is a positive correlation between sensitivity to the probability of PasSub’s winning and subjects extraversion. c) positive correlation between subjects’ other-regarding behavior in the loss condition and their belief about others’ cooperation in Public Goods Game (PGG).

Eqs 1 to 3 show the proposed computational model. The mean fitted standardized parameters with their SEM are shown in Fig 5. The sixth parameter, temperature, which shows randomness in the choice, is not shown in Fig 5 for better illustration (mean = 170.99, SEM = 17.61 (95%)). The mean self-regarding parameter is positive when subjects are in the “Lose” conditions, and the mean other-regarding parameter is slightly negative. As the model suggests, when subjects win, their other-regarding behavior increases and their self-regarding behavior decreases on average. However, the net value of these changes (self-regarding motives change+other-regarding motives change) is negative, and since self and other-regarding parameters are in alignment together in our task, it leads subjects to share less. In addition, on average, subjects have a negative tendency toward the value of information; they share less when the probability of winning for the other person increases, i.e., their information is more valuable. In Fig 5, the model parameters have been calculated for the subject with high variance in their behavior since we did not have enough data for the subject at the two ends of the sharing spectrum (almost always sharing subjects or rarely sharing subjects) to fit the model. Therefore we had outliers in the model’s parameters when we tried to fit the model for these subjects. However, the patterns in the model parameters remain the same without removing the low variance subjects (for the criterion of selecting always sharing subjects and rarely sharing subjects, see S1 File, subjects types).

Furthermore, there is a significant positive correlation between the model parameter *w*_*information*_ and subjects extraversion (Pearson correlation, R = 0.46, p-value = 0.0019); the more subjects are extravert, the easier they give high probability information. Also, the subjects’ other-regarding behavior in the loss condition in the computational model is positively correlated with their belief about others’ level of cooperation in the Public Goods Game (Pearson correlation, R = 0.48, p-value = 0.012). We observed a negative correlation between their other-regarding parameter in the win condition and their extraversion (Pearson correlation, R = -0.41, p-value = 0.0057).

## Discussions

We live in an era where information-sharing is one of the most prominent means to remain connected with others. Although most of the shared information has no impact on our choices, i.e., categorized as non-instrumental information, we share more than billions of messages [1] on social networks on a daily basis. Also, experimental researches show that we eagerly seek and share such information [15, 29]. The pandemic of sharing useless information suggests that such information has no monetary value for its distributors. Nevertheless, sometimes our personally useless information can be of monetary usage to others. Getting aware of this fact, do we still freely share our personally useless and short-lifetime information?

We investigated sharing personally useless information behavior when others can benefit from it in an information-sharing task. In our task, the subject first faced an n-choice reward-seeking problem and either found the goal and got a reward or failed and received nothing. Then, the subject gained some personally useless trial-specific information (short-lifetime information); complete or partial information about the goal position in the win and lose conditions, respectively. The subject could share that information with her anonymous confederate (who would face the same n-choice reward-seeking problem) and get a defined percentage (0, 20 or 50 percentages) of her confederate’s reward in that trial.

The first observation was that the subjects’ behavior was substantially different from the common passionate free-information-sharing one. Although information sharing was cost-free and beneficial for their confederates in almost 90 percent of the trials, the subjects did not show altruistic behavior. The surprising point was that the subjects shared less when their confederates would benefit the most.

Sharing information was not only cost-free but also was potentially beneficial for the beholders in 2/3 of the trials. Therefore sharing even in all conditions, could be rationalized in terms of expected reward maximization and decision load minimization. However, some of the subjects were highly inclined toward not sharing; unexpectedly, they preferred to waste their personally useless and short-lifetime information by not sharing it. More importantly, some of the subjects did not share even in the seemingly fair division of outcome, i.e., 50% of the benefit.

In order to explain these surprising results, we proposed a computational model based on self and other-regarding behavior and the subjective value of information. Furthermore, the model assumes that subjects’ self and other-regarding motives are not fixed, i.e., they can change by inducing emotions in the win condition. In terms of model recovery, the model had good prediction at the general level as well as in each condition. Besides, the parameter recovery for the model was acceptable (see S1 File, parameter recovery).

Based on this model, subjects can be reluctant to share information if their other-regarding motive or their sensitivity to the value of information (how much the subject increases her confederate’s winning probability) is negative. On the other hand, the model predicts sharing more if their selfishness and prosocial behavior are positive.

Interestingly, according to model-free analysis (subject’s sensitivity to benefit percentage) and computational modeling (sensitivity to the value of information parameter), the subjects assumed a significant value for their personally useless information. The more the value of this information was, the less subjects wanted to share. These findings support the view that even personally useless and very short-lifetime information has value in subjects’ minds [29–32]. However, one might assert that as the value of information (probability of reward for both ActSub and PasSub) increases, the subjects willingness to share information decreases simply because they do not want to seize the PasSub’s reward for themselves. For example, consider sharing in the win condition as an extreme example that the ActSub knows that if she shares, an amount of PasSub’s money will be seized for her. Hence, since ActSub care about other’s outcomes, they decrease their willingness to share. Here, we explain that this assertation is less likely by bringing up two reasons. First, if the subjects were hesitant toward sharing just because they did not want to seize PasSub’s earnings, then, we would expect the subjects to share more in 0% condition (relative to 20% or 50%) which is not the case (Fig 2). Second, the subjects sensitivity to reward probability was correlated with their level of extraversion. That is in favor of regarding this parameter as value of information instead of hesitation to seize the PasSub’s reward. This also aligns with the observation that introverts shared less.

When the subjects lost, selfishness was the main drive for sharing. In contrast, other-regarding did not have a significant effect on sharing in the lose condition (mean = -0.25, SEM = 0.25, two-sided t-test, p-value = 0.32, between subjects analysis). Therefore, the subjects shared more when their expected profit from sharing compensated for their subjective value of information better and avoided sharing their personally useless information otherwise despite being expired. In other words, the result of computational modeling rationalizes the observed monetary irrational behavior. That is, high personal monetary benefit is needed to compensate for low other-regarding motives and high subjective value for personally useless and fast-expiring information. It suggests cognitive interventions for decreasing the subjective value of information when monetary compensation for sharing is limited or costly.

The subjects had certain information just in their personal win trials, and in those trials, they shared less; compared to probabilistic information cases. This is surprising since we expect the subjects to share more as sharing certain information leaves no doubt in their confederates’ win, and consequently, their own benefit is guaranteed. The higher tendency to avoid sharing can be attributed to both satisfactions from self-win followed by a change in behavior or to the nature of sharing certain information, i.e., giving a free lunch to others. If certain information had an independent role in preventing subjects from sharing, then we should expect a significant change in the value of information in the win condition. In order to test this, we added another parameter to our computational model named “change in the value of information”. The model parameters pattern remained the same as the former model, and the “change in the value of information” was not significantly different from zero (mean = 0.09, SEM = 0.19, two-sided t-test, p-value = 0.65, between subjects analysis). It means that certain information did not have a role different from the role of information value. Instead, the results pointed at a significant drop in the self-regarding motive as the possible cause of lower sharing in the win condition.

One possible explanation for the change in self and other-regarding motives might be related to incidental emotions, i.e., emotions that are not associated with the actual choice problem, claiming that happy emotions can increase people’s prosocial behavior and decrease their selfishness [33–35]. Therefore, we expect the self (other) regarding parameter to decrease (increase) in the win situation, which was the case in our computational modeling. However, one may expect an increase in sharing information when subjects become more prosocial and less selfish, which is in contrast with our behavioral results; the subjects shared less when they won. We resolve this contradiction by looking at the subjects’ behavior as well as the model parameters and the task structure. The task structure aligns selfish and other-regarding motives together; sharing always helps the subjects and their confederates (except in zero benefit when sharing only helps the confederate). Therefore, the net value of the decrease in selfishness (negative impact on sharing) and an increase in prosocial behavior (positive impact on sharing) change the sharing performance. That net value was negative in our win cases; i.e., the drop in selfishness was significantly larger than the increase in prosocial motives. This observation suggests that inducing happy emotions does not necessarily increase individuals’ monetary contribution in society when they benefit directly or indirectly from their contributions. Nonetheless, further studies are needed in order to investigate whether this effect has occurred because of happy emotions.

Sharing information was positively correlated with the subjects’ level of extraversion. According to the modeling results, the subjects with low levels of extraversion had more negative sensitivity to the value of information; introverts shared less when the information value was higher, while extraverts were not sensitive to the information value. One possible explanation for this correlation is that more extravert subjects might be less reluctant to give information. Therefore, in their mind, there is no difference between sharing high probability versus low probability information. However, further investigations are needed to study this hypothesis.

We observed that sharing was not linked to the fairness of opportunities nor the subjects’ level of cooperation. Some subjects had negative sensitivity to the fairness of opportunities, excluding certain information and zero benefit percentage cases. However, the results were not generalizable to the whole population.

Experimental results showed that sharing in the zero benefit condition and the subjects’ belief about other’s cooperation in the public goods game were positively correlated. Our modeling linked this behavior to a higher other-regarding parameter; there was a positive correlation between the other-regarding parameter and the subjects’ beliefs about others’ cooperation in the public goods game. These observations corroborate the idea that subjects’ beliefs about others’ cooperativeness can be the mediator of their other-regarding behavior [23, 24]. This observation is interesting because the subjects played with anonymous people in PGG who did not have anything to do with the information-sharing task. Thus, this correlation is important in way that it shows us that the way that people think about others’ cooperation in their surroundings affects their attitude towards others in general and that could influence their behavior in any social interaction, including information-sharing. In other words, the level of one’s optimism toward society’s cooperation can affect their altruism in other specific interactions. We also observed a significant correlation between the subjects’ other-regarding parameter in the win condition and their extraversion that needs to be explored more in future studies.

The GLM results revealed that around 15% (50%) of the subjects were biased toward sharing (not sharing) their information, while no significant bias was observed in 35% of the subjects. This heterogeneity in the automaticity of sharing decisions suggests that, in contrast to Tamir et al. ‘s claim [15], sharing information has not been an intrinsic behavior in most of the subjects in our setting.

One may argue that the subjects had been sensitive to their own and their confederates’ accumulated rewards. We added these two parameters to the model to examine this, and that assumption was rejected. The same happened to the difference between these accumulated rewards. The reason would be either lack of strong competitiveness or not calculating the accumulated reward by the subjects. In addition, we ruled out the role of other retrospective factors such as envy, regret, and guilt of the previous trial.

The current study has some possible extensions. First, the underlying neural mechanism of information sharing in our task is unclear. Tamir et al. [15] used an information-sharing task in which subjects had to give up money to inform others and used fMRI techniques to investigate the underlying associated brain areas during the information-sharing process. They found that informing others is intrinsic and is associated with the reward circuits. Interestingly, in our non-costly information-sharing environment, we observed that some of the subjects shared highly with relatively short reaction times while others refused to share their information again with relatively short reaction times. This observation was further supported by the correlation analysis between the utility functions in the computational model and the reaction times. The difference between the utility of sharing and not-sharing negatively correlated with the difference between the mean reaction time of the shared and not-shared trials (corr = -0.60 and p-value<0.0000001); see Hutcherson et al. [22] for a similar observation. Hence, it is important to study the underlying brain mechanism of the subject’s behavior at the two ends of the sharing spectrum to see whether sharing information for those who almost never share is intrinsic or not.

Furthermore, personally useless information and money conceptually have some similarities since they are only valuable in social interactions. That being said, we should be aware that we lose our money by sharing it, contrary to information. Therefore, one may expect a higher tendency to share information relative to money. It could be worth comparing money allocation with information sharing framings and investigating the role of costs and intrinsic information sharing as well as their underlying neural mechanisms.

Cost of the information is another area that can be studied in future research lines. For example, imagine that you have a fading-out research idea with some preliminary but not yet publishable results and do not have funding or human resources to pursue it in due time. How do you rationalize not sharing your idea with another colleague in return for mutual scientific collaboration and benefit? In addition to the previously mentioned factors that prevent subjects from sharing (low other-regarding motives and considering a high value for the information), subjects might not share their research information in these situations (or any kind of information in other situations) simply because the process of transferring knowledge and ideas and in general the communication are costly [18]. Hence, it is important to study the role of costs and its interaction with other motives.

Using anonymous subjects also helped us prevent social dynamics’ confounding effects. That being said, there are many situations where subjects share their information to build up a good public image [1]. This effect can be added to our model as a bias to the benefit percentage. Hence, since the main driver for sharing information was selfish motives, one may expect that subjects share more information when publicly visible and not anonymized. However, it is unclear whether the subjects care about their image in front of others, equally as their monetary benefit. Also, our self-regarding and other-regarding motives might change in the presence of relational dynamics. This effect should be investigated more deeply in future studies.

Finally, it is unclear whether nature or nurture has caused the intrinsic biases toward giving or not giving the information. Further research is needed to investigate whether intrinsic biases are related to genetics or not. In parallel, cross-cultural studies are required to investigate social preferences toward sharing personally useless information among different cultures [36].

### Details of analysis

All analysis were conducted with MATLAB software, version 2015a.

### RT analysis

We removed RTs that were more than 10 seconds in order to make the shape of the logarithm of RT distribution of all subjects normal. 36 trials out of 17500 trials were removed. The mean RT before removing these trials was 1.51s, and the mean RT after removing these trials became 1.48s. We also excluded five subjects in the t-test analysis since they had less than ten shared or not-shared trials in order to conduct a t-test to compare shared and non-shared trials. The results are robust to the selection of this minimum number of trials in shared and not-shared decisions.

## Supporting information

S1 File (DOCX)
