# Supplementary material for: Do you share your personally useless information if others may benefit from it?
Source: PLoS One. 2022 Oct 17;17(10):e0276062. doi: 10.1371/journal.pone.0276062 (PMC9576053; doi:10.1371/journal.pone.0276062)
Supplement: S1 File — (DOCX) [file pone.0276062.s001.docx]

**Do you share your personally useless information if others may benefit from it?**

Aryan Yazdanpanah^1^* , Abdol-Hossein Vahabie^1, 2, 3^ , Majid Nili Ahmadabadi^1^*

1. Cognitive Systems Laboratory, Control and Intelligent Processing Center of Excellence (CIPCE), School of Electrical and Computer Engineering, College of Engineering, University of Tehran, Tehran, Iran
2. Department of Psychology, Faculty of Psychology and Education, University of Tehran, Tehran, Iran
3. School of Cognitive Sciences, Institute for Research in Fundamental Sciences, Tehran, Iran

*Corresponding authors: Majid Nili Ahmadabadi ([mnili@ut.ac.ir](mailto:mnili@ut.ac.ir)), Abdol-Hossein Vahabie (h.vahabie@ut.ac.ir)

**Supplementary Materials**

**This PDF file includes:**

Materials and Methods

Supplementary Results

Table of Contents:

1. Materials and methods
   1. Additional tasks
      1. Public Goods Game
      2. Big5 personality traits
   2. Computational modeling
      1. different models of social preference
      2. three variants of the selected model
      3. criteria for selecting the subjects’ model parameters
2. Supplementary results
   1. Subjects types
   2. regression analysis
      1. general factors
      2. Envy of previous trial, guilt, and regret
   3. Reaction time analysis
   4. parameter recovery

**I. Materials and methods**

a. Additional tasks

i. Public goods game

30 subjects of those who previously participated in our main task participated in PGG study one year and a half after the task. The task was a one-shot game with 4 participants. Each participant was given 8 thousand Tomans in Iranian currency (~80 cents) to play with in the game. The task was taken internet-based. The money the subjects allocated to the public good, were doubled and shared between participants equally. After finishing the task, we asked the subjects about their beliefs on how much other three participants has shared in the game. Furthermore, we asked them two questions to be sure they have understood the game.

ii. personality traits

We took Big 5 personality traits questionnaire one year after task completion. Since some of the subjects were not available anymore after this time, we could collect the data from 53 of those 70 subjects participated in our first study. If we assume that the subjects’ personality traits do not have huge changes in one year, the subjects behavior in our first task and their personality traits must be correlated in case sharing personally useless information is a personality trait.

b. computational modeling

i. different models of social preference

**Computational model**

A simple utility-based model can be built upon the utility that ActSub expects from sharing information:

$U(\mathrm{share})=\mathrm{P}(\mathrm{win}_{PasSub}|\mathrm{share})\times\mathrm{U}_{benefit}(\gamma\times\mathrm{reward}_{PasSub'ssuccess})$ (1)

Where$U(\mathrm{share})$is the expected utility of sharing,$P(\mathrm{win}_{PasSub}|\mathrm{share})$is the PasSub’s win probability if ActSub shares information, $U_{benefit}$is ActSub’s utility of its confederate’s reward when$\gamma$is the benefit percentage. In this modeling, $U(\mathrm{Not}\mathrm{Share})=0$. Hereafter we use identity function for $U_{benefit}$. The decision-making model is a Boltzmann machine:

$\Pr(\mathrm{share})=\frac{e^{U(\mathrm{Share})/\mathrm{T}}}{e^{U(\mathrm{Share})/\mathrm{T}}+e^{U(\mathrm{Not}\mathrm{share})/\mathrm{T}}}$(2)

A pure expected utility maximizer is anticipated to share when $U(\mathrm{share})>0$. However, since$\gamma\geq0$, $P(\mathrm{share})=1$ not only results in expected benefit maximization but also reduces the decision making load. T is the temperature and is fitted during model fitting process.

In this model, the active subject does not consider any social value and is assumed to be selfish. However, widely used models of social preferences include confederate’s expected utility as well.

**The Fehr-Schmidt model**(*16*)

This model is based on inequity aversion:

$\begin{aligned} U(share)=U_{ActSub}(share)-\beta\times max\{0,U_{ActSub}(share)-U_{PasSub}(share)\} \\ -\alpha\times max\{0,U_{PasSub}(share)-U_{ActSub}(share)\} \end{aligned}$ (3)

where $\alpha\geq\beta$ and $0\leq\beta<1$

$U_{ActSub}(\mathrm{share})=\mathrm{P}(\mathrm{win}_{PasSub}|\mathrm{share})\times(\gamma\times\mathrm{reward}_{PasSub'ssuccess})$ (4)

$U_{PasSub}(\mathrm{share})=\mathrm{P}(\mathrm{win}_{PasSub}|\mathrm{share})\times((1-\gamma)\times\mathrm{reward}_{PasSub'\mathrm{s}success})$ (5)

$U(\mathrm{Not}\mathrm{Share})$ is calculated likewise by setting$\gamma=0$, and replacing $P(\mathrm{win}_{PasSub}|\mathrm{share})$ by$P(\mathrm{win}_{PasSub}|\mathrm{not}\mathrm{share})$in (4) and (5).

**Bolton-Ockenfels model**(*45*)

This model is also relying on inequity aversion. However, the sensitivity to inequity is measured by a single parameter in contrast with the previous model:

$U(share)=U_{ActSub}(share)-\beta{(\frac{U_{ActSub}(share)}{U_{ActSub}(share)+U_{PasSub}(share)}-\frac{1}{2})}^{2}$(6)

Other variable and parameters are defined as the same as the previous model.

**Krajbich et al. model**(*50*)

This model is based on how much a person cares about her confederate, and it is represented in the model by the parameter$\alpha$. The formulation is as the following:

$U(share)=U_{ActSub}(share)+\alpha U_{PasSub}(share)$(7)

It is obvious that if the parameter $\alpha$ is negative, the player 1 cares negatively about player 2 and vice versa for the condition in which $\alpha$ is positive. Other conditions are defined as the same as the previous models.

**Hutcherson et al. model**(*26*)

This model is also based on how much a person cares about the other person. However, they have added another degree of freedom to the model that represents how much a person cares about herself:

$U(share)=W_{self}U_{ActSub}(share)+W_{other}U_{PasSub}(share)$(8)

Similar to the previous models, other conditions are defined.

Initially, we fitted these models to our data and selected the best model (Hutcherson et al.’s model) in terms of mean BIC.

ii. three variants of the selected model

After selecting the winning model, we used model prediction to see if Hutcherson et al.’s model could predict the behavior or not. The result showed that the model could not capture subjects’ behavior solely, especially in the win conditions. Therefore, before inserting information value to the model, we used examined three hypotheses about the mechanistic model of changing behavior in the win condition.

1. win condition as a bias in the model

In this version, we assumed that the win condition is an independent psychological phenomenon and affects subjects utility of sharing as the following:

$U\left( share \right)=W_{self}U_{ActSub}\left( share \right)+W_{other}U_{PasSub}\left( share \right)+ W_{other}\times Win$ (9)

In this model, Win is a dummy variable and equals to 1 if the subject has won and is zero otherwise.

1. win condition changes the ActSub payoff

In this version, the utility for share and not share decisions is calculated as the equation (8). However, there is a difference in calculating the $U_{ActSub}(share)$:

$U_{ActSub}\left( share \right)=P\left( win_{PasSub} | share \right)\times\left( \gamma\times reward_{PasSub^{'s}success} \right)+$ $Win\times reward_{ActSub^{'s}success}$ (10)

Win is a dummy variable similar to the former version.

1. win condition changes subjects self and other-regarding motives

In this variant of the model, we changed the self and other-regarding parameters in the share and not share utilities as the following:

(11)

The results showed that the last version that win condition changes subjects self and other-regarding motives fits better to the data. Therefore, we continued with this model.

iii. criteria for selecting the subjects’ model parameters

Since some subjects had a low variance behavior in their sharing intentions, i.e., almost always shared or almost never shared, we could not have an accurate fitting of the parameters in these clusters (see supplementary results, subjects’ types). Therefore, for calculating correlations between model parameters and Big5 (or PGG behavior), and reporting the model parameters, we removed the subjects with low variance in their behavior (standard deviation less than 0.2), based on the subjects’ standard deviation histogram and the individuals’ standard deviations depicted on figure below.

Figure S1 Standard deviation of subjects (vertical axis) vs. subjects sharing intentions. The colors represent different clusters of sharing behavior that has been explained in the next part. Standard deviation less than 0.2 removes dark blue (almost always share) and black (almost never share) clusters only.

**ii. Supplementary results**

1. **Subjects’ types**

Clustering the subjects

To find similarity and diversity in the subjects’ behavior, we calculated the percentage of shared trials in each of the 15 conditions and used these 15 features to cluster the subjects. Since the number of features was large in comparison with our sample size (70 subjects), we used a hierarchical clustering method. The algorithm used for computing the distance between clusters was unweighted average distance, and the distance metric was cityblock. We had four single-member clusters, which we considered them as outlier clusters in the clustering section.

Fig. S2 shows the average frequency of sharing for all subjects in 15 information sharing conditions. Now the question is if the subjects can be clustered with respect to their sharing behavior. The hierarchical clustering resulted in the formation of 6 distinct groups; see Fig. S2a. The characteristic behavior of the groups can be described as:

1. Almost never share information (3males, 8 females)

2. Negative sensitivity to information gain and interest in higher benefit percentage (12 males, 8 females)

3. Negative attitude to provide certain win condition and interest in higher benefit percentage (8 males, 1 female)

4. Mostly share information except in zero benefit condition (4 males, 7 females)

5. Mostly share information except in certain win condition (3 males, 2 females)

6. Mostly share information (4 males, 6 females)

**Figure S2 a Hierarchical clustering. Ten clusters are formed, and 4 of the clusters were single-member ones. The rest 6 clusters are analyzed. b The average percentage of shared responses is shown with details for the biggest cluster. We had three different benefit percentages for ActSub (0%, 20%, and 50%) and for each benefit percentage we had five different IGs. Two of these IGs are win conditions, and 3 of them are the conditions in which the ActSub has failed. The IGs are sorted for win and lose conditions separately. In this cluster, the percentage of sharing decreases with increasing the IG. c Percentage of each cluster in the population.**

b. regression analysis

**GLM model**

In our GLM model, we first included the general factors in our task structure, then, we added parameters such as guilt, envy of previous trial and regret to our model separately.

1. General factors

$U_{diff}=\beta_{0}+\beta_{1}s+\beta_{2}\log(\frac{p_{2}}{p_{1}})+\beta_{3}z$(10)

In the proposed model, the probability of sharing is calculated from the following formula:

$\Pr(\mathrm{share})=\frac{e^{U_{diff}}}{1+e^{U_{diff}}}$(11)

The parameter$\beta_{0}$represents the intrinsic biases of subjects about sharing information. Parameter$\beta_{1}$measures sensitivity to ActSub’s benefit percentage and parameters$\beta_{2}$and$\beta_{3}$are sensitivity to IG and sensitivity to win conditions respectively. In this model, $Z$ is one for win conditions and zero otherwise, $S$can be 0, 0.2, or 0.5 and$\log(\mathrm{P}_{2}/\mathrm{P}_{1})$can be derived from the conditions mentioned in Fig. 1b.(in the main text) It is worth to be mentioned that we used different combinations of variables $p_{1}$and$p_{2}$ in our model such as linear combination, multiply, and the ratio of $p_{2}$to $p_{1}$but the best-fitted model was the model that used$\log(\mathrm{P}_{2}/\mathrm{P}_{1})$. It should be noted that variables $Z$and $\log(\mathrm{P}_{2}/\mathrm{P}_{1})$have a strong correlation. In order to eliminate the effect of correlation between these two variables, we used the Gram-Schmidt orthogonalization method and used the orthogonalized vector of$\log(\mathrm{P}_{2}/\mathrm{P}_{1})$. The parameters of modeling were very close to the former parameters. Thus, we continued without orthogonalization.

As can be seen, the intercept of the model increases with increasing the tendency of information sharing. If we define this intercept as subjects’ intentions toward sharing information, almost 50% of the subjects have negative intentions. Parameter $\beta_{1}$corresponds to subjects’ sensitivity to the percentage of benefit. There is not any subject with negative sensitivity to the percentage of benefit, and almost 75% of the subjects have positive sensitivity. It means that the subjects are more willing to share their information if they get more. The parameter $\beta_{2}$represents the subjects’ sensitivity to the information gain (IG) or fairness of opportunities. Most subjects are not sensitive to IG, but some have negative sensitivity; that means their tendency of sharing information decreases if IG increases. The subjects with significant sensitivity to IG are mostly laid on the left part of X-axis in fig. S3, where the sharing tendency is lower. The last parameter,$\beta_{3}$, is the subjects’ sensitivity to win conditions and is negative in almost 40% of the subjects; they decide to share less when they win and have certain information for their confederate.

**Figure S3 GLM parameters are fitted for each individual and have been normalized to their standard error. The horizontal axis is the tendency of sharing information and the vertical axis in each figure is one of our model’s parameters. Each data point represents an individual, and its color and sign correspond to its cluster. The subjects with model parameters below/above the lower/higher red lines have negative/positive sensitivity to that variable. As it can be seen for$\beta_{1}$, no individual has negative sensitivity to the percentage of benefit.**

1. envy of previous trial, guilt, and regret

In our GLM model, we added the conditions in which the behavior of subject in the previous trial, might have had an effect on the current trial in the form of guilt, regret, or envy. These retrospective variables were equal to 1 if there was such a condition in the previous trial and were equal to zero otherwise. We added these variables separately to the GLM model since they had correlation with each other and couldn’t be added to the model simultaneously. In this kind of retrospective analysis, if a subject has not shared the information in the trial n-1 and PasSub has failed, the subject might have guilt (because of not sharing) and this may affect her behavior in the trial n. In this analysis, we first assessed subjects’ sensitivities to guilt and then calculated the correlation between sensitivity to guilt and the tendency of sharing information. The analysis showed that there is not a significant correlation between retrospective guilt and tendency of sharing information (corr=0.19, p-value=0.10). In the same analysis, we calculated the subjects’ sensitivities to regret. Again, in this retrospective context, subjects’ behavior in trial n-1 might lead them to be regretful in trial n, if they have not shared, PasSub has won, and benefit percentage has not been zero in trial n-1. In other words, if they could have won something but they haven’t because they have not shared in the previous trial, they must be regretful. There was not a significant correlation between subjects’ sensitivity to regret and their tendency of sharing information (corr=-0.13, p-value=0.29). Furthermore, from Fehr-Schmidt model, envy arises if the payoff of the other person gets more than the payoff of self. From this point of view, retrospective envy happens when PasSub’s payoff has been more than ActSub’s payoff in the previous trial. However, our analysis again showed that retrospective envy is not correlated with sharing personally useless information (corr=-0.08, p-value=0.50).

1. **Reaction time Analysis**

We calculated each ActSub’s mean RT in shared and not-shared trials; see Fig. S4a. The horizontal axis represents the frequency of sharing information in all trials. According to Fig. S4b, there is a significant difference between RTs in shared and not-shared trials at the two ends of the sharing behavior spectrum; i.e., for those who either rarely (cluster 1) or mostly (cluster 6) share. Those who preferred to keep/share their useless information were faster in their not-shared/shared decisions. That is, sharing/not-sharing is the faster decision for those who mostly/rarely contribute their information. In contrast, there was no significant difference in RTs of shared and not-shared decisions of the other subjects; those who were more sensitive to the benefit percentage and the information gain. In addition, those subjects had a longer RT in both their shared and not-shared decisions, compared to the faster decisions of mostly and rarely sharing subjects.

**Figure S4 a mean RT in shared and not-shared conditions. The horizontal axis is the percentage of shared information in 250 trials for each subject. Each dot represents a subject, and its color and sign indicate its cluster. Outliers are represented with multiplication signs. The five dots with red circles around them are the subjects who have less than ten trials of one of the shared or not-shared responses, and as a result, they are not included in a t-test. b t-statistics for the difference between mean RT in shared responses and mean RT in not-shared responses. Outliers are represented with multiplication signs. Note that below the lower red line, the mean RT for not-shared responses is significantly greater than mean RT for shared responses. The pattern is reversed for the data above the upper red line.**

1. **parameter recovery for the proposed model**

After finding the low variance subjects, we did the parameter recovery for the subjects who had a noticeable variance in their behavior. Figure S5, shows these subjects.

**Figure S5 parameter recovery for the model parameters. The vertical axis is the** **recovered parameters and the horizontal axis is the fitted ones.**
